# A *Picea abies* Linkage Map Based on SNP Markers Identifies QTLs for Four Aspects of Resistance to *Heterobasidion parviporum* Infection

**DOI:** 10.1371/journal.pone.0101049

**Published:** 2014-07-18

**Authors:** Mårten Lind, Thomas Källman, Jun Chen, Xiao-Fei Ma, Jean Bousquet, Michele Morgante, Giusi Zaina, Bo Karlsson, Malin Elfstrand, Martin Lascoux, Jan Stenlid

**Affiliations:** 1 Department of Forest Mycology and Plant Pathology, Swedish University of Agricultural Sciences, Uppsala, Sweden; 2 Department of Ecology and Genetics, Evolutionary Biology Centre, Uppsala University, Uppsala, Sweden; 3 Institute for Systems and Integrative Biology, Université Laval, Québec City, Québec, Canada; 4 Dipartimento di Scienze Agrarie e Ambientali, Universita di Udine, Udine, Italy; 5 Skogforsk, Svalöv, Sweden; China Agricultural University, China

## Abstract

A consensus linkage map of *Picea abies*, an economically important conifer, was constructed based on the segregation of 686 SNP markers in a F_1_ progeny population consisting of 247 individuals. The total length of 1889.2 cM covered 96.5% of the estimated genome length and comprised 12 large linkage groups, corresponding to the number of haploid *P. abies* chromosomes. The sizes of the groups (from 5.9 to 9.9% of the total map length) correlated well with previous estimates of chromosome sizes (from 5.8 to 10.8% of total genome size). Any locus in the genome has a 97% probability to be within 10 cM from a mapped marker, which makes the map suited for QTL mapping. Infecting the progeny trees with the root rot pathogen *Heterobasidion parviporum* allowed for mapping of four different resistance traits: lesion length at the inoculation site, fungal spread within the sapwood, exclusion of the pathogen from the host after initial infection, and ability to prevent the infection from establishing at all. These four traits were associated with two, four, four and three QTL regions respectively of which none overlapped between the traits. Each QTL explained between 4.6 and 10.1% of the respective traits phenotypic variation. Although the QTL regions contain many more genes than the ones represented by the SNP markers, at least four markers within the confidence intervals originated from genes with known function in conifer defence; a leucoanthocyanidine reductase, which has previously been shown to upregulate during *H. parviporum* infection, and three intermediates of the lignification process; a hydroxycinnamoyl CoA shikimate/quinate hydroxycinnamoyltransferase, a 4-coumarate CoA ligase, and a R2R3-MYB transcription factor.

## Introduction

Norway spruce [*Picea abies* (L.) Karst.] is ecologically one of the most important conifer species in Europe, naturally present in central Europe across the Alps, the Carpathians and the Balkans, and in northern Europe from the west coast of Norway to far into the Russian mainland [1]. It is also one of the most important species economically in European and Swedish forestry, constituting 41% of the standing Swedish tree volume [2]. This dominance means that vast areas of forest land are largely monocultural, covered by only *P. abies*. Albeit favouring a single species has been a sound strategy in terms of generating forest revenue, this approach has also been extremely beneficial to the forest pathogen *Heterobasidion parviporum,* which is a highly competitive early colonizer of fresh wounds and newly cut stumps. *H. parviporum* is a causal agent of annosum root rot, a serious and very common fungal disease in conifer forests of the Northern Hemisphere [3] causing yearly losses to European forest owners exceeding € 790 million yearly in growth reduction and devaluation of timber [4]. In 1986, the average incidence of root rot in Swedish Norway spruce trees was estimated to 15% [5], and the frequency has later been reported to increase in managed forests with ∼23% per decade [6].

Trees less susceptible to root rot would be highly coveted by the forest industry. Nevertheless, most breeding endeavours so far have been directed at high tree growth, with little or no effort targeted at breeding for trees resistant to *H. parviporum* infection. Still, resistance breeding is potentially fruitful, as it has been shown that *H. parviporum* resistance is a genetically variable trait and does not affect growth rate negatively [7]. It would be an important step towards efficient resistance breeding to have molecular resistance-associated markers as well as better knowledge of whether resistance is a multifactorial trait or instead, mainly controlled by a single locus. One way to shed light on this is through quantitative trait loci (QTL) mapping of *P. abies* resistance to *H. parviporum* infection.

Mapping of QTLs in genomes such as those of conifers of the *Pinaceae* family is fraught with various problems. The huge size of the *Pinaceae* genomes alone (∼14–37 Gb) [8]–[9] has its drawbacks, but the extremely low gene density in combination with great regions of repeatable elements with low recombination rate [10] is an even greater obstacle in terms of converting identified QTLs to underlying sequence variation. In *P. abies*, the haploid genome is estimated to 19.6 Gb and the number of transcribed genes to 28,354 [11], making the average distance between two neighbouring genes 0.69 Mb. The proportion of various repetitive elements in the genome has been estimated to 70% [11], making whole genome assembly a difficult task where very few, if indeed any, scaffolds are likely to be large enough to contain more than a single gene. This makes it very hard to know the genome sequence between two given *P. abies* loci. In terms of making sense out of a QTL, this means that only markers originating from a known position are of potential use; anonymous markers are almost impossible to translate unless they originate from the same scaffold as a sequence-tagged marker. It also means that if several markers within a QTL are significantly associated with the trait, only the ones originating from the gene actually controlling the trait will carry any immediately useful information. This is a significant limitation compared to QTLs in maps based on less fragmented genome sequences since the search for potential candidate genes is more likely to be rewarding if the entire QTL is located to a single, large scaffold. On the other hand, the low gene density also means that there will be a limited number of possible candidate genes in the vicinity of each marker associated with the trait. Despite these potential obstacles, QTL analyses have been applied previously to map host resistance in similar pathosystems. For example, *Pinus radiata* resistance to Dothistroma needle blight has been found to be controlled by multiple QTLs [12], and two major QTLs explained 52% of the variance in *Eucalyptus globulus* resistance to *Mycosphaerella cryptica* infection [13]. Furthermore, Eucalyptus inter-specific hybrids have been used to show a multifactorial control of resistance to *Puccinia psidii* rust infection [14], and rust resistance in both *Populus* and *Salix* spp. is based on several loci [15]–[16].

Several linkage maps of *P. abies* have been constructed already, but only one [17]–[18] has been saturated enough to create a number of linkage groups corresponding to the haploid number of chromosomes. However, of the 768 markers in that map, only 32 were derived from ESTPs and thus originating from known genes, crucial for making use of QTLs in fragmented genome assemblies. Another *Picea* map, based on a *Picea mariana* x *Picea rubens* hybrid population, consisted of 835 positioned markers, of which 318 originated from known genes [19]. In terms of mapped genes, the most comprehensive conifer maps to date has been constructed for *Picea glauca* [20], containing 1743 SNP markers from transcribed genes, and twice for *Pinus taeda* [21]–[22], based on 2841 and 2393 segregating genes.

The goal of this study was to map resistance QTLs in *P. abies* involved in response to four aspects of *H. parviporum* infection. The map was constructed using 247 full-sib progenies from a cross between two *P. abies* parents, based on the segregation of 686 polymorphic SNP markers, all derived from known *P. abies* genes. All four traits were controlled by multiple loci, explaining between 4.6 and 10.1% of the phenotypic variance in the mapping population. If the effect of all loci is additive, the variation within the four traits would be explained by in total 12–25.7%. These are the first resistance QTLs identified against this fungus, and also the first identified against any pathogen in *P. abies*. Naturally, the SNPs underlying these QTLs only represent a fraction of the actual genes and might not be the ones causal for the traits. Nevertheless, the SNPs originated from genes with homologs to at least four plausible candidate genes. One of these, a leucoanthocyanidine reductase, has previously been shown to be upregulated in *P. abies* in response to *H. parviporum* infection [23].

## Materials and Methods

### The mapping population and resistance assays

The biological material used to construct the linkage map and conduct the resistance assays was a family of originally 251 progenies (later adjusted to 247), with six ramet cuttings from each original ortet, from a cross between the *Picea abies* parents S21K7622162 and S21K7621678. The biological material and the virulence assays have been described previously [24]. In short, the resistance of the progeny was estimated by infecting four ramets of each progeny, as 2-year-old potted plants, with a *Heterobasidion parviporum* (strain Rb 175) infested wooden plug through a 5×5 mm cambial wound. The entire assay was conducted in the greenhouse. After 4 weeks, death of the inner bark (lesion) was noted and stems were cut aseptically into 5-mm lengths that were incubated in moist condition. Sapwood growth of *H. parviporum* was assessed from the presence of conidiophores on sections of the stem. These data were treated as best linear unbiased prediction (BLUP) values [24]. Resistance was estimated by measuring lesion lengths in the bark surrounding the wound, fungal growth within sapwood up- and down-stem from the wound and fungal exclusion, i.e. the ability of the host to exclude the fungus once it has entered the sapwood. The fungal exclusion concept was based on the observation that after 4 weeks infection, the fungus was not always continuously present in the sapwood all the way from the wound to its farthest frontline but rather seemed to lose substrate to the host, whose ability to reclaim sapwood thus can be seen as a resistance trait. Exclusion was calculated as the cleansed proportion of the infected sapwood: exclusion  =  x/y, where x is the distance of fungus-free sapwood between the wound and the fungus, and y is the total distance of sapwood between the wound and the fungal front. Both x and y were calculated as mean values across all four ramets.

Arnerup et al [24] observed no fungal material in 27% of the infected plants and argued that this might either be due to infections failing at an early stage and never reaching the sapwood, or due to a later, full exclusion of the infection (x  =  y). They further observed that excluding or including the potentially failed infections had little effect on the overall results. To investigate whether these 27% could be genetically explained as the ability of the host to stop the fungus from entering the wound altogether, this was also mapped as a separate trait. Infection prevention was quantified as the proportion of ramets of each progeny that did not contain any conidiophores after incubation and had no more than 2 mm inner bark lesion up- or down-stem from the wound after 4 weeks of infection.

### DNA extraction and SNP marker design

Genomic DNA was extracted from spruce needles of the two ramets remaining from each progeny after the resistance assays, using the DNeasy Plant Mini Kit (QIAGEN, Germantown, MD). An Illumina 3072 SNP Golden Gate Assay was developed by merging SNPs from a number of different resequencing and genotyping projects. All markers included in the assay had a score higher than 0.6 in the Illumina OPA design. The majority of SNPs (1879) were originally identified in and designed for *Picea glauca* and later also tested on and found to be variable in a small number of *P. abies* individuals [25]. The rest of the SNPs came from three different sources; 250 from a previously developed 768 SNP Golden Gate Assay [26], 269 from sequencing pooled PCR products using Illumina next-generation sequencing technology (Ma XF, Zaina, G, Källman T, Chen J, Morgante M, Lascoux M, unpublished), and 674 SNPs identified in a single individual subjected to mRNA sequencing using Illumina technology [27].

### Linkage analysis and map construction

The Illumina Golden Gate assay was used to genotype the mapping population according to standard procedures [28] at the SNP Technology Platform, Uppsala University (http://molmed.medsci.uu.se/SNP+SEQ+Technology+Platform/Genotyping/). The SNP markers formed three clearly distinguishable clusters (A/A, A/a, a/a), and those polymorphic between the parental trees were used as mapping data. Their segregation patterns within the 247 progenies were observed and analysed using JoinMap 3.0 (Kyazma) [29] and visualized using MapChart 2.1 (Plant Research International) [30]. To be accepted, a marker had to be scored as present or absent (i.e., not as missing data) in at least 80% of the individuals. Markers with a segregation pattern deviating strongly from the expected ratio (*P*<0.005, χ^2^) were also omitted. The segregation data were coded as CP, i.e. a population resulting from a cross between two heterogeneously heterozygous and homozygous diploid parents. Linkage groups were determined using pairwise comparisons at minimum likelihood of odds (LOD) value of 4 and a recombination frequency threshold 0.4. As no previous information on marker order existed, the internal order within groups was accepted as presented by the Map 2-function (regression mapping, Kosambi's mapping function, adding markers one at a time, accepting the position that results in the best goodness-of-fit for the map, discarding markers that results in a *jump* in χ^2^ for goodness-of-fit of 5 or more), after discarding any markers that contributed to the *mean* χ^2^ for for goodness-of-fit of the group by more than 4. Finally, to screen for double recombination events, an average genotype probability (–Log10(P)) threshold value of 0.3 was set for each group, eliminating all markers with a higher average. Similarly, markers involved in highly improbable genotypes (-Log10(P) >2.0) in more than 10 of the 247 individuals were also removed.

From each gene carrying more than one marker, every marker but the best genotyped one (defined by least missing data and least distorted segregation) was deemed as redundant. Markers were deemed as originating from the same genes as another marker, if they shared identical accession numbers in GenBank, TAIR or the *Picea glauca* genome and mapped within 10 cM of each other. Even though recombination in *P. abies*, as in many plants, seem to occur mainly within the genes [31]–[33], the population of the present study is not big enough and the SNP markers not numerous enough to expect many recombination events between these redundant markers. As they are expected to have identical genotypes, the discordance can be used as a way of measuring genotyping error in the data. The effect of this genotyping error was estimated by summing the largest possible distance between two markers on every gene with redundant markers and divide by the number of such genes. The QTL ranges were subsequently adjusted at both ends by this mean in order to compensate for the genotyping error.

Genome length was estimated using method 4 of Chakravarti *et al.* [34], in which the observed length in cM of each linkage group is multiplied by (m +1)/(m –1), with m being the marker count for each group. Genome coverage was calculated as (total observed genome length)/(total estimated genome length), and as the proportion c of the genome within d cM of a marker, using the formula c = 1 – e^-2dn/L^ [35] with L being the estimated genome length and n the number of mapped markers. As these methods assume normal distribution of data, we tested for this by dividing every linkage group into 10 cM fragments and counting the number of markers, x, in each, with x ranging from 0 to 15. A Poisson distribution function, *P*(x)  =  µ^x^e^-µ^/x!, was used to compare this to a normal distribution, with *P*(x) being the probability of x markers per 10 cM fragment and µ the genome-wise average number of markers per fragment. The expected distribution of intervals with x markers was calculated by multiplying *P*(x) for each x (0–15) with the total number of 10 cM intervals in the map. Finally, a Kolmogorov-Smirnov test for two populations was conducted to decide whether the observed distribution of markers was likely similar to the expected distribution [36].

### QTL analyses

BLUP values for lesion lengths and fungal growth in sapwood, and mean values for excluded proportion of total infection length [24], were used in the QTL analyses, whereas raw proportion data was used for the infection prevention trait.

The likelihood of resistance QTLs was determined using the MapQTL (Kyazma) mixture model method [37] according to the following approach. First, interval mapping [38] (200 iterations) was employed for every 1 cM throughout the linkage map in order to identify markers significantly associated with the trait. The significance was calculated by a permutation test (*P*<0.05, χ^2^) (5000 permutations). QTL peaks significant at either genome or linkage group level were picked for further study. Then, the marker closest to the respective regional QTL peak was tentatively picked as cofactor and tested using the restricted MQM-mapping algorithm (as there were multiple putative QTLs for each trait). If this altered the position of the QTL peak, a new cofactor was picked. This process was repeated until every designated cofactor was located as closely as possible to the peak of their respective QTL. The confidence interval for each resulting QTL was determined as a decrease in 1 LOD unit on both sides of the QTL LOD-peak, adjusted at both ends by the mean effect of the suspected genotyping error (see above). Finally, a Kruskal-Wallis test was conducted to determine the individual level of association between the markers within the confidence interval and the examined traits. A QTL was only deemed significant if it contained at least one significant marker according to the Kruskal-Wallis test (*P*<0.05, marker level). Such markers were noted as potentially causative for the trait along with the designated cofactor (Table 1). All calculations were performed using MapQTL 4.0 (Kyazma) [37].

**Table 1:** Numeric data and SNP content for each resistance QTL found in the *Picea abies* linkage map.

Trait	QTL interval at -1LOD (cM)			LOD thresholds P<0.05. χ2							
Linkage group	Interval	LOD peak (cofactor peak)	Cofactor	% explained^1^ (at cofactor)	Linkage group level	Genome level	SNPs in QTL interval	KW Significance^2^	Position	Accession/TAIR number	BlastX homology^3^
Infection prevention											**hydroxycinnamoyl CoA shikimate/**
1	152.4–158.8	3.95	GQ03113-N13.1.1005	5.3 (5.1)	3.2	4.4	08pg07937c	0.05	158.4	BT101292	**quinate hydroxycinnamoyltransferase**
							GQ03113-N13.1.1005	0.05	158.6	BT107879	uncharacterized protein
2	64.0–82.5	3.37 (3.33)	FCL324Contig1-740	5.9 (5.8)	3.1	4.4	GQ03010-E07.1.207	0.05	69.9	BT106774	5-alpha-reductase
							FCL324Contig1-740	-	73.5	AT3G54260.1	TBL-gene family member
11	96.2–119.4	3.83 (3.68)	PabiesFT1-1251	7.2 (6)	3.0	4.4	208PG15708n	0.005	96.8	BT100755	**pectin methylesterase**
							PabiesFT1-1251	0.01	114.2	BT115191	Mother of FT-like protein
							GQ0255-K05.2.1102	0.05	114.3	BT103501	**R2R3-MYB transcription factor MYB11**
Exclusion											
1	157.5–161.8	3.59 (3.53)	02776-N18-3098	4.8 (4.6)	3.2	7.6	FCL3309Contig1-1061	0.05	161.3	AT3G44730.1	kinesin-like protein 1
							02776-N18-3098	0.01	161.3	AT1G09160.1	protein phosphatase 2C
2	93.7–99.8	3.31 (3.31)	GQ0041-H19.1.1034	5.1 (5.1)	3.1	7.6	FCL1145Contig1-703	0.05	96.5	AT5G50920.1	ATP-dependent Clp protease ATP-binding subunit/ClpC
							GQ0041-H19.1.1034		97.7	BT100742	Cu/Zn Superoxide dismutase
3	74.4–80.1	3.96 (3.96)	GQ04004-O18.1.247	5.7 (5.3)	3.2	7.6	GQ04004-O18.1.247	0.0005	75.7	BT118309	**4-coumarate:CoA ligase**
6	133.4–158.9	5.24 (5.23)	GQ02803-C08.1.1246	10.1 (8)	3.2	7.6	GQ03404-I14.1.173	0.005	136.3	BT113308	uncharacterized protein
							GQ03302-B08.1.275	0.005	137.1	BT111676	uncharacterized protein
							GQ02803-C08.1.1246	0.005	140.3	BT103859	uncharacterized protein
							GQ03412-L23.1.1340	0.05	144.9	BT113543	Elongation factor Tu, chloroplastic
Lesion length											
8	0.0–5.2	5.28 (5.15)	0-9749-01-9969-co	6.7 (6.2)	2.8	4.0	0-12329-01-11666-	0.01	0.0	AT1G49760.2	polyadenylate-binding protein
							0-9749-01-9969-co		0.9	AT5G49760.1	**Leucine-rich repeat protein kinase family protein**
							02739-B22-309	0.01	4.2	AT4G21450.3	PapD-like superfamily protein
9	32.9–61.7	3.64 (3.64)	NODE-3044-length-2	5.3 (5)	2.9	4.0	NODE-3044-length-2	0.05	47.3	PUT-175a-Picea_glauca-12549270	uncharacterized protein
Growth in sapwood											
2	135.8–142.7	3.42 (3.42)	GQ02827-E23.1.1092	4.6 (4.2)	3.1	4.3	GQ02827-E23.1.1092	0.05	141.2	BT105733	short chain dehydrogenase
											
6	42.9–53.0	5.79 (5.75)	WS-2.0-GQ0064.T	8.4 (8.1)	3.2	4.3	WS-2.0-GQ0064.T	0.005	46.3	BT105286	uncharacterized protein
	176.8–182.6	3.54 (3.54)	GQ03204-B13.1.1304	4.8 (4.8)	3.2	4.3	GQ03719-P11.1.1591	0.05	178.8	BT116508	magnesium-protoporphyrin IX monomethyl ester [oxidative] cyclase, chloroplastic [Vitis vinifera]
							GQ03204-B13.1.1304	0.01	182.6	BT109050	**leucoanthocyanidin reductase**
9	103.7–113.1	5.27 (5.27)	PabiesFT4pr-2046	7.3 (7)	3.1	4.3	PabiesFT4pr-2046	0.005	110.4	BT112861	flowering locus T-like/terminal flower1-like protein
							GQ03615-J11.1.673	0.05	112.0	BT115393	G2/mitotic-specific cyclin
							0276-A13-934	0.01	112.9	AT2G36970.1	UDP-glycosyltransferase superfamily

1 Percentage of phenotypic variation explained by this QTL at its LOD peak. In parenthesis the percentage explained at the cofactor.

2 Significance of association according to Kruskal-Wallis-test. 0.05 = *P*<0.05, χ^2^

3 **Bold** indicates homology (BlastX, MaxScore >378, E-value < 1e-125) to Pinaceae genes, *Italics* homology (BlastX, MaxScore >83.2, E-value < 1e-15) to angiosperm genes, with a suggested role in defence response

After the significant QTLs were identified, the false discovery rate (FDR) [39] was estimated in order to determine the rate of type I errors (i.e. false discoveries). The individual *P*-values for every significant QTL, for the individual traits as well as all pooled together, was listed as *P*(1), P(2) … *P*(m), where *P*(1) was the smallest and *P*(m) the largest. According to the Benjamini-Hochberg (BH) procedure [39], the FDR is controlled at the specified significance level q if *P*(i) < q*i/m, where *P*(i) is the *P*-value of a given QTL in the list, i is the numerical listed position of that QTL (i.e., between 1 and 13 for the pooled traits, since 13 QTLs were found) and m is the number of QTLs considered.

## Results

### Polymorphic SNP markers segregating in the progeny

Screening the 247 progenies and the parental trees S21K7622162 and S21K7621678 against the Illumina 3072 SNP Golden Gate Assay discovered that 1014 assays failed to produce useful results. This resulted in 2058 (67%) successfully scored SNP markers. Of these, 874 (43%) SNPs proved to be polymorphic among the parental genomes and useful as genetic markers. The marker names, sequences and corresponding accession numbers in GenBank, TAIR and the *Picea glauca* genome are presented in Table S1. Analysing the SNP markers revealed that the 874 markers originated from 769 unique transcribed genes. Sixty-nine of these genes gave rise to more than one SNP marker each. In total, 174 of the 874 SNP markers came from such multi-SNP generating genes, i.e. the dataset had 105 redundant markers. The most widely spaced redundant markers of each corresponding gene mapped on average 0.97 cM of each other. Since not enough recombination events should occur within these genes to justify this figure, redundant markers were removed so that each of the 769 transcribed genes were represented by only one SNP marker.After further trimming the marker set by removing those that were heavily distorted (*P*<0.005, χ^2^) or poorly characterized (missing data in >20% of the population), 738 markers remained. Of these, 292 SNPs were heterozygous in S21K7622162 (segregating 1∶1), 309 in S21K7621678 (1∶1), and 137 were heterozygous in both parents (segregating 1∶2∶1) (Table 2). Thirty-five SNP markers (4.7%) segregated at a ratio distorted from the expected 1∶2∶1 (7 markers) or 1∶1 ratio (28 markers) (0.005<*P*<0.05, χ^2^).

**Table 2:** Statistics for SNP markers segregating between the parental trees S21K76221 and S21K7621678.

	Heterozygous for both parents	for S21K7622162	for S21K7621678	Total
Polymorphic^1^	137	292	309	738
Distorted (0.005<*P*<0.05)	7	16	12	35
Positioned	118	278	290	686
Unlinked	19	14	19	52

1 Remaining after removing heavily distorted loci (P<0.005) or missing data in >20% of the mapping populatio

The SNP analysis showed that eight individuals (four pairs) of the originally 251 members of the mapping population were between 97.5% and 99.3% identical in terms of SNP genotypes. As this probably was due to DNA contamination at some stage, one member of each pair was omitted, resulting in 247 trees.

### Twelve well supported linkage groups

JoinMap and MapQTL input files are included as Tables S2 and S3.

**Figure 1:**
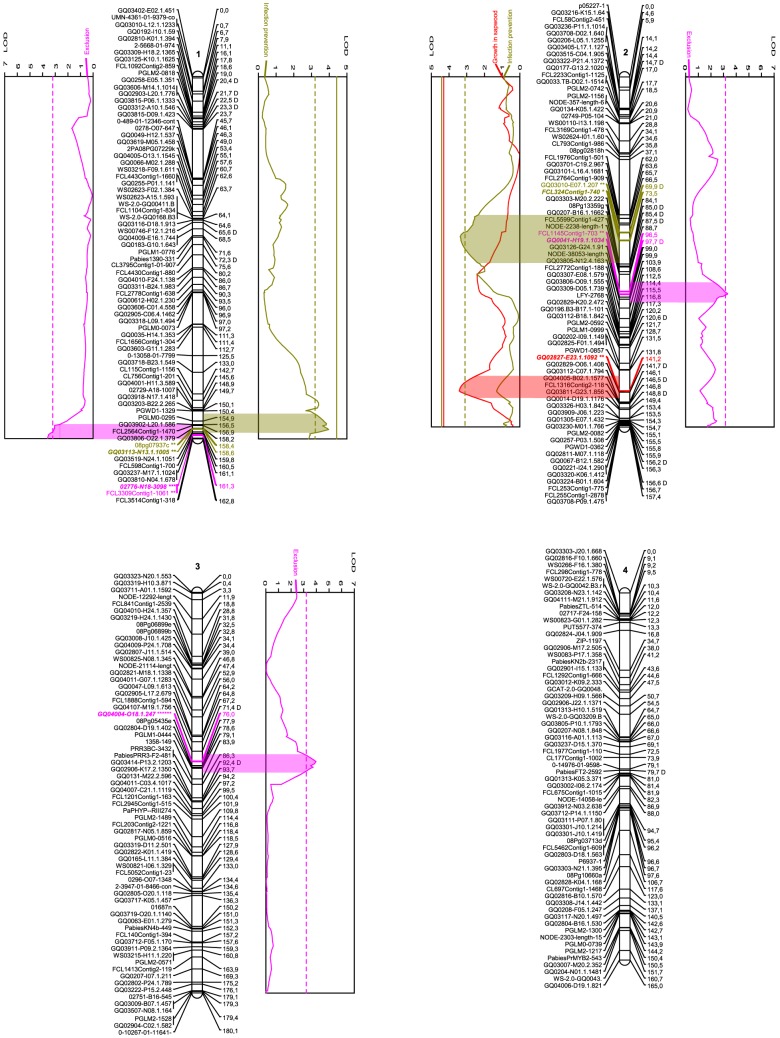
Linkage groups 1-4 of the *Picea abies* genome and the QTLs for various resistance traits. Names of the SNP markers are displayed on the left of the linkage groups. Genetic distance (cM) is indicated on the right. SNP markers are described in detail in Table S1. Graphs denote QTL effects for the current group. Red curves indicate fungal growth within sapwood, purple fungal exclusion and orange curves indicates infection prevention. Complete and dashed vertical lines describe 0.1% and 5% levels of significance for the individual trait and group. Wide colored areas between curve and group show the QTL confidence interval based on a 1 LOD drop from the QTL peak. The colored marker denotes SNPs under the QTL confidence interval, the stars level of significance according to the Kruskal-Wallis test (ranging from p<0.1 (*) to p<0.0005 (******) and **bold** style the designated cofactors. “D” after a marker name indicates segregation pattern deviating from the expected Mendelian ratios of 1∶1 or 1∶2∶1 (0.005<*P*<0.05, χ^2^). Image created using MapChart 2.1 (Plant Research International). [28]

**Figure 2:**
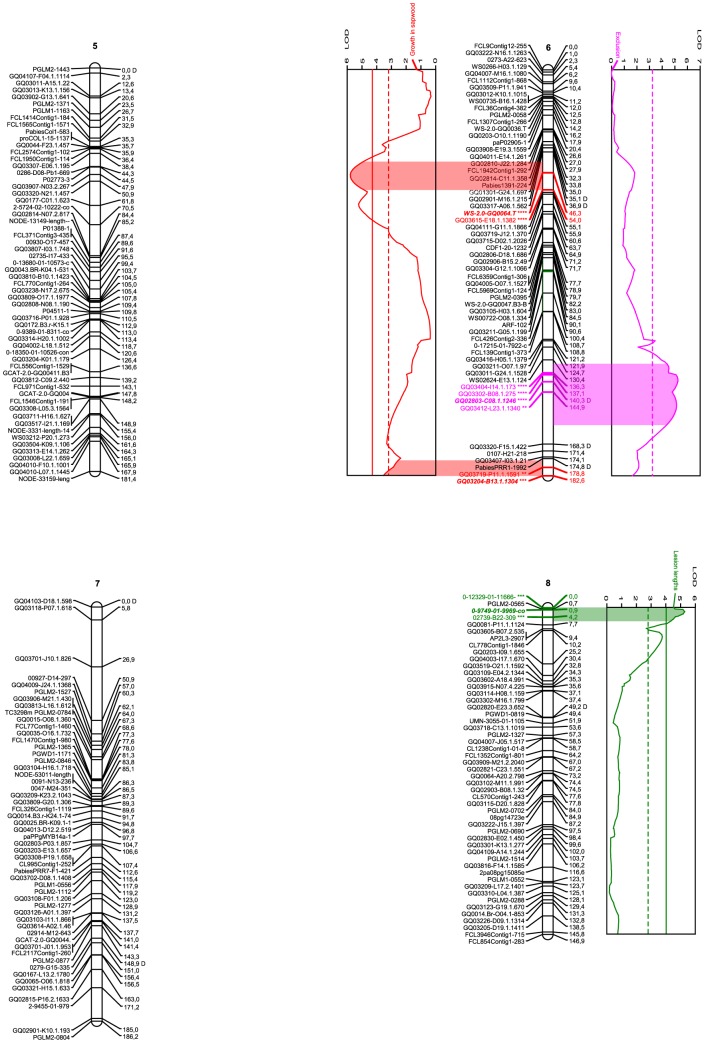
Linkage groups 5-8 of the *Picea abies* genome and the QTLs for various resistance traits. Names of the SNP markers are displayed on the left of the linkage groups. Genetic distance (cM) is indicated on the right. SNP markers are described in detail in Table S1. Graphs denote QTL effects for the current group. Red curves indicate fungal growth within sapwood, green lesion length, and purple fungal exclusion. Complete and dashed vertical lines describe 0.1% and 5% levels of significance for the individual trait and group. Wide colored areas between curve and group show the QTL confidence interval based on a 1 LOD drop from the QTL peak. The colored marker denotes SNPs under the QTL confidence interval, the stars level of significance according to the Kruskal-Wallis test (ranging from p<0.05 (**) to p<0.005 (****) and **bold** style the designated cofactors. “D” after a marker name indicates segregation pattern deviating from the expected Mendelian ratios of 1∶1 or 1∶2∶1 (0.005<*P*<0.05, χ^2^). Image created using MapChart 2.1 (Plant Research International). [28]

**Figure 3:**
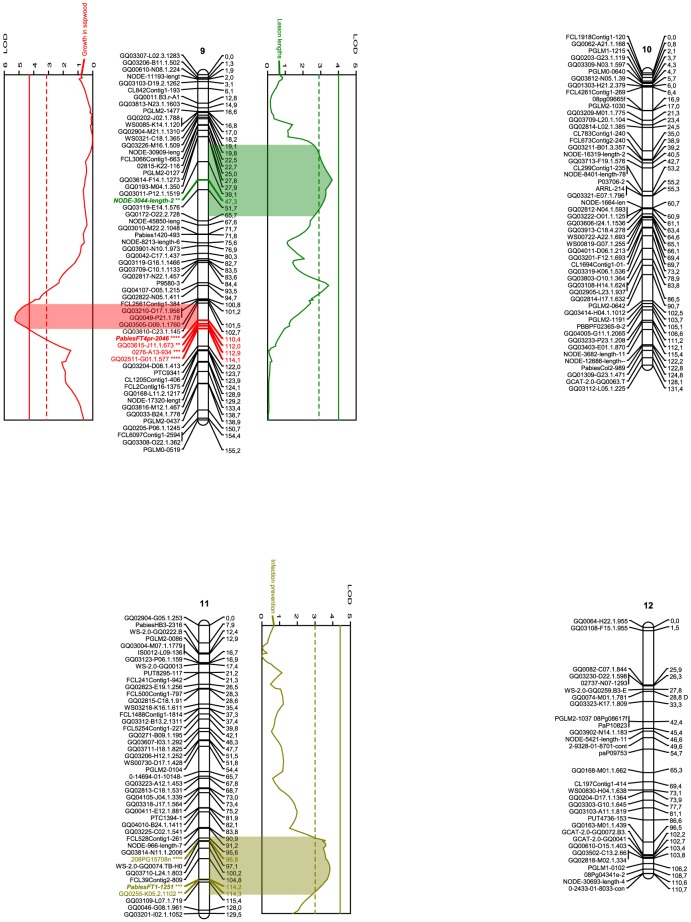
Linkage groups 9-12 of the *Picea abies* genome and the QTLs for various resistance traits. Names of the SNP markers are displayed on the left of the linkage groups. Genetic distance (cM) is indicated on the right. SNP markers are described in detail in Table S1. Graphs denote QTL effects for the current group. Red curves indicate fungal growth within sapwood, green lesion length, and orange curves indicates infection prevention. Complete and dashed vertical lines describe 0.1% and 5% levels of significance for the individual trait and group. Wide colored areas between curve and group show the QTL confidence interval based on a 1 LOD drop from the QTL peak. The colored marker denotes SNPs under the QTL confidence interval, the stars level of significance according to the Kruskal-Wallis test (ranging from p<0.05 (**) to p<0.005 (****) and **bold** style the designated cofactors. “D” after a marker name indicates segregation pattern deviating from the expected Mendelian ratios of 1∶1 or 1∶2∶1 (0.005<*P*<0.05, χ^2^). Image created using MapChart 2.1 (Plant Research International). [28]

The markers that proved to be involved in many improbable genotypes (average –Log10(P) for linkage with neighbouring markers above 0.3, or –Log10(P) above 2.0 in more than 10 individuals of the population) were deemed suspicious and omitted during the mapping process. After this final screening, which removed 20 markers, JoinMap 3.0 (Kyazma) organized 686 of the remaining 718 into a consensus map of twelve linkage groups (Figures 1-3) as per result of the Map 2-function. No marker in the resulting linkage groups contributed with more than 4 to the mean χ^2^ of goodness-of-fit for the group. Although a minimum LOD score of 4 was arbitrarily determined as sufficient to accept a linkage group as significant, this threshold was not relevant as all twelve groups remained stable up to a LOD score of 10. The groups varied in size from 110.7 to 186.2 cM and included from 32 to 73 markers (Figures 1-3, Table 3). The twelve linkage groups covered 1889.2 cM in total and had an average density of 2.8 cM/marker, varying across the groups from 2.2 to 3.5 cM/marker. The genome wide physical size to genomic distance ratio was 10.4 MB/cM, if assuming the Norway spruce genome to be 19.6 GB large [11]. Across the groups, assuming each chromosome is 1.63 GB large, the ratio varied between 8.8 and 14.7 MB/cM (Table 3).

**Table 3:** Numeric data for each linkage group in the *Picea abies* linkage map.

Linkage group	Size (cM)	SNP markers. total	SNP markers. distorted segregation	cM/marker	Linkage group size (% of total length)	Chromosome size (% of total length)^1^	Chromosome size (% of total length)^2^	∼Mb/cM^3^
1	162.8	72	7	2.3	8.6	8.7	8.6	10.0
2	157.4	73	12	2.2	8.3	8.6	8.1	10.4
3	180.1	67	2	2.7	9.5	9.0	9.1	9.1
4	165.0	64	1	2.6	8.7	8.8	8.9	9.9
5	181.4	60	1	3.0	9.6	9.3	9.7	9.0
6	182.6	59	5	3.1	9.7	9.3	9.8	8.9
7	186.2	54	2	3.4	9.9	10.7	10.8	8.8
8	146.9	51	1	2.9	7.8	7.4	7.5	11.1
9	155.2	58	0	2.7	8.2	8.2	7.8	10.5
10	131.4	52	0	2.5	7.0	7.0	7.3	12.4
11	129.5	44	0	2.9	6.9	6.9	6.7	12.6
12	110.7	32	1	3.5	5.9	6.0	5.8	14.7
Avg	157.4	57.2	2.7	-	8.3	8.3	8.3	-
Total	1889.2	686	32	2.8	100	100	100	10.4

1 Relative chromosome size according to Lubaretz, 1996.

2 Relative chromosome size according to Siljak-Yakovlev, 2002.

3 Calculated on an average chromosome size of 1.63 Gb (Nystedt, 2013).

**Figure 4:**
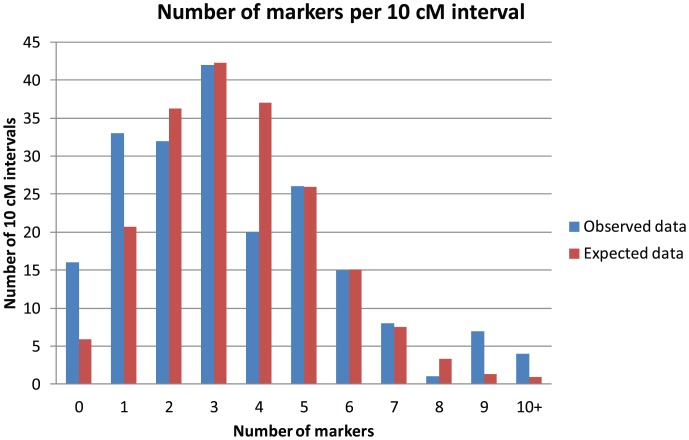
Distribution of markers. Observed and expected Poisson distribution of the markers frequencies for each 10

Genome length was estimated as 1958 cM, using the (m +1)/(m – 1) method [34]. The map thus covers 96.5% of the estimated genome and has a 97.0% probability that any given locus in the genome lies at most 10 cM from a positioned marker (determined from c – see Material and Methods). Whether the observed distribution of markers across 10 cM blocks of the genome was different from the expected random distribution was tested with a Kolmogorov-Smirnov test. The difference was small (D = 0.08) and non significant (P = 0.53). The distributions are displayed in Figure 4.

Of the 35 markers displaying distorted segregation, 3 were unlinked while 32 (91.4%) were distributed over 9 of the 12 the linkage groups (Table 3). Six groups had just 1 or 2 distorted markers, while the other three groups (1, 2 and 6) had 7, 12 and 5 markers, respectively. In three cases two or more neighbouring distorted markers constitute a cluster. On LG 1, five distorted markers were positioned between 20.4 and 23.3 cM, whereas on LG 2, three others were clustered between 84.9 and 87.4 cM. Two distorted neighbouring markers were found on LG 6 (at 35.1 and 36.9 cM), while the other 14 on these groups were distributed without any other such marker as their closest neighbour.

### Resistance variation detected in the population

**Figure 5:**
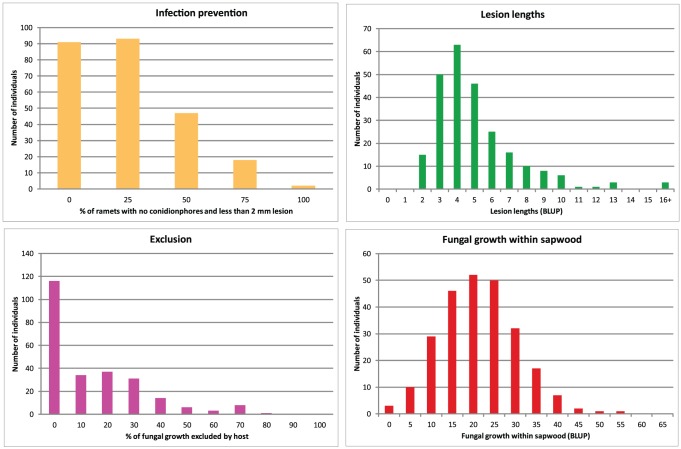
Distribution of resistance data. The distribution of observed resistance, measured as lesion length, fungal growth within sapwood, fungal exclusion, and ability to prevent infection throughout the mapping population, using all four ramets.

Two-hundred and fifty-one full sibling plants stemming from a cross between the *P. abies* parents S21K7622162 and S21K7621678 were used for scoring resistance against *H. parviporum* infection. The resistance variation among the progeny, expressed as lesion lengths around the wound and fungal growth within the sapwood, has been described previously [24]. In addition, the exclusion of fungus from the host was calculated as the proportion of fungal growth in sapwood eliminated from the wound and outward, and the ability to prevent infection was calculated as the proportion of ramets completely lacking conidiophores after 4 weeks of infection. The distributions of the resistance over the progeny for all traits are displayed in Figure 5.

### Resistance mapping reveal multiple QTLs

As visible from Figures 1-3 and Table 1, the traits exclusion (purple in Figures 1–3), lesion length (green), growth in sapwood (red) and infection prevention (orange), were associated with four, two, four, and three specific QTL regions, as per defined by a 1-LOD interval around a significant QTL LOD-peak (permutation test, *P*<0.05, χ^2^), containing at least one marker significant at *P*<0.05, χ^2^ according to the Kruskal-Wallis test, and adjusted at both ends by 0.97 cM in order to compensate for genotyping error. The QTL regions contained nine, four, seven, and seven such SNP markers, respectively. The QTLs for each trait explained similar percentages of the phenotypic variation; 4.8–10.1% for the exclusion (25.7% in total, if additive), 5.3–6.7% for the lesion lengths (12% in total), 4.6–8.4% for the growth in sapwood (25.1% in total), and 5.3–7.2% for the infection prevention (18.4% in total), while the significant (*P*<0.05, χ^2^) LOD peaks varied between 3.31 and 5.79 across the traits and QTLs. Three QTLs (1 for lesion lengths, 2 for growth in sapwood) were significant at the genome level; the others only at the chromosome level. The genes corresponding to each significant SNP and the corresponding cofactor can be seen in Table 1.

The BH-procedure [39] showed that for the QTLs from individual traits as well as for all QTLs in the study together, the false discovery rate was controlled at q = 0.05, since *P*(i)<q*i/m for every i (QTL) at that level of significance (see Material and Methods).

Among these resulting 27 SNP markers, we identified four markers in genes with homology (BlastX, MaxScore >378, E-value < 1e-125) to *Pinaceae* genes with a suggested role in defense response: a hydroxycinnamoyl CoA shikimate/quinate hydroxycinnamoyltransferase [40], a 4-coumarate CoA-ligase [41], a R2R3-MYB transcription factor [42], a leucoanthocyanidine reductase [43], (Table 1). These are specifically involved in the phenylpropanoid and flavanoid pathways.

## Discussion

One of the purposes of QTL mapping is to investigate the number of involved loci in complex traits and their individual importance. Another coveted benefit of QTL mapping is the identification of candidate genes behind these traits, which makes it essential to be able to decipher the mapped area into actual sequence. Because of the difficulties in assembling conifer DNA into scaffolds containing multiple genes, the number of known neighbouring genes to any given identified gene in the *Picea abies* genome is virtually zero. Genetic linkage can circumvent this obstacle, but only if the markers are fashioned from known sequence, i.e. not anonymous. An example of such markers is SNPs located in transcribed genes. Since the *P. abies* whole transcriptome has been sequenced and a large part of the genome assembled [11], and transcriptome profiles are available for *P. glauca* and *P. sitchensis* [44], the potential for SNP based linkage maps to identify causative genes for important traits is growing. Extreme high-density maps containing gene-based markers for every single gene seem possible in the foreseeable future.

In the present study, a full-sib *P. abies* family of 247 progenies, stemming from a cross between the parents S21K7622162 and S21K7621678 [24], was used to construct a consensus linkage map based on segregation patterns of 686 SNP markers. The markers were derived from an Illumina 3072 SNP Golden Gate Assay, resulting in 2058 successful assays and 874 informative markers. The success rate from SNP design to successful SNP core (67%) was consistent with what has been reported from conifers earlier [19], [45]–[47]. The final linkage map is one of the most saturated *P. abies* linkage map to date and the one most enriched in mapped genes, although well behind some other conifer maps such as the 1745 genes mapped in *Picea glauca* [20] or the 2841 in *Pinus taeda* [22]. Quantitative trait loci for four distinct traits of resistance against *Heterobasidion parviporum* infection were positioned on the map. This is the first report of resistance QTLs in any host to this economically important pathogen, which is a vital step towards the identification of the causal genes.

The twelve linkage groups of the present map correspond to the twelve chromosomes of *P. abies* and most other species of the *Pinaceae* family [48]. The linkage groups are all large, varying between 110.7 and 186.2 cM and between 32 and 73 unique loci. As visible from Table 3, this variation in size among the linkage groups corresponds to a relative size of 5.9 to 9.9% of the total map length. This mimics the variation in relative size among *P. abies* chromosomes, which span from 6.0 to 10.7% and 5.8 to 10.8% of the total genome size as per two previous estimations based on morphometrics of karyotype data [49]–[50]. Of course, this correlation is only circumstantial evidence that the largest linkage group reflects the largest chromosome, but still suggests not only that the linkage groups indeed represent the actual chromosomes, but also that the SNP markers have been derived from all parts of the genome. This assumption was strengthened by the fact that the map covers 96.5% of the estimated genome length, and that every loci is with 97% probability located within 10 cM of a mapped marker. Thus, the map should be saturated enough to allow QTL detection for every causative region for the respective trait, and even though adding more markers to the map certainly would make the existing groups denser, it would not substantially increase the map size in centimorgans. This assumption has been confirmed in a saturated *Picea glauca* map [20], which was increased from 1301 to 2211 markers, while decreasing from 2086.8 to 2065 centimorgans.

There is strong evidence that the marker organisation into groups is valid, since every group remained stable up to LOD 10 and no marker had an average genotype probability (–Log10(P)) higher than 0.3. In total, the twelve groups span 1889.2 cM, with a marker density of 2.8 cM/marker. This is fairly similar to the only other *P. abies* map of similar saturation available [17]–[18], which reported a size of 2035 cM and 2.6 cM/marker using 775 markers. As expected, the 174 SNPs sharing a mutual origin with another marker always did map very close to their intragenic neighbours - the average distance between such markers (measured on the most widely spaced two of each gene) was 0.97 cM. Recombination in *P. abies*, and plants in general, has indeed been suggested to occur mainly within genes [31]–[33], but for enough such events to occur within these genes to justify this average distance, the genes would have to be many times larger than the genome average. Instead, this might reflect the genotyping error rate for the experiment. Knowing that two of markers that were expected to locate very close to the same position actually were separated by on average 0.97 cM infers that every marker position in the map should be considered as potentially being misplaced by this much. To compensate for this, the QTL confidence interval defined by the 1-LOD drop was adjusted by 0.97 cM.

Markers exhibiting segregation distortion can be a sign of erroneous genotyping data, especially if the markers are randomly dispersed across the map and do not aggregate into clusters [51]. On the other hand, clusters of distorted markers may weaken map structure [52]. In this study, 4.7% (32) of the 686 positioned SNP markers segregated at a ratio distorted from the expected Mendelian ratios of 1∶1 or 1∶2∶1 (0.005<*P*<0.05, χ^2^). This proportion is similar to the 6% reported for a *P. abies* map previously [17]–[18], but lower than the 12% reported for a *P. mariana* x *P. rubens* cross [52] and higher than the 1.9% reported for *P. glauca* [18]. As for other conifers, similar distortion levels have been reported for maps of *Pinus* species, such as 9% for *P. sylvestris* [53], 1–2.4% for various populations of *P. pinaster* [54], 7.4–7.5% for two pedigrees of *P. taeda* (*P*<0.005, χ^2^) [55] and 12% for a *P. palustris* x *P. elliottii* cross [56], while 5.4% of 1364 markers has been reported as distorted (*P*<0.01, χ^2^) in *Cryptomeria japonica* [57]. In this study, ten distorted markers formed three minor clusters (i.e. had at least one distorted immediate neighbour) while the other 22 were scattered across the map (had no such neighbour). However, since the distortion level was not extreme (0.005<*P*<0.05, χ^2^) and their presence did not force any other markers out of the groups, they were included.

When distorted markers are clustered, the reason may be selection bias. As certain genotypes are lethal or otherwise detrimental to viability, there will be an inherent selection for these areas, so-called viability QTLs. Viability QTLs are of course interesting in their own right as they may provide insight into genes with a pivotal impact on fitness, but they are not necessarily involved in the biology of host resistance. Without selection, the segregation pattern in a family would be expected to follow Mendelian ratios all across the genome. At a viability QTL, these ratios would be distorted due to the genotype-dependent mortality [58]. Also, viability QTLs would be expected to be significant for all measurements, as the effect is based on selection prior to phenotyping. It is not likely that the QTLs of this study can be considered as viability QTLs, because only three of the 27 SNP markers found within a QTL confidence intervals in this study were distorted (*P*<0.05, χ^2^), all in different QTLs, on two different linkage groups (2, 2 and 6), and none of the QTLs were significant for more than one measurement.

The finalized map was used to locate QTLs for four phenotypically distinct aspects of resistance against *H. parviporum* infection using interval mapping [38]. Interval mapping assumes that the measured traits follow a normal distribution across the mapping population. As visible from Figure 5, at least the exclusion and infection prevention traits do not follow a normal distribution but rather seem to have a spike at 0 effect (a null phenotype). Interval mapping can still provide reliable results, if the spike at 0 is small and the rest of the data set normally distributed and not much larger than 0 [59]. This is probably not the case of the exclusion and infection prevention traits. In order to verify the QTLs observed for these traits, a Kruskal-Wallis test was performed. A Kruskal-Wallis test is a non-parametric test that makes no assumption about the probability distributions of the traits, and it was performed on each locus separately without using any linkage information [37]. The standpoint was that loci deemed significant both by interval mapping and the Kruskal-Wallis test would be considered as valid QTLs even if the data was not normally distributed. This approach was also employed for the normally distributed traits for Lesion length and Growth in sapwood (Figure 5).

To avoid scoring false positive QTLs while at the same time maintaining enough statistical power to detect as many true positive QTLs as possible, the false discovery rate (FDR) was estimated at the level of significance q. According to [60], all traits in a multitrait study should be tested simultaneously in a FDR-controlling approach. The BH-test [39] was used both for all traits together and for each trait individually. For both approaches, the FDR was controlled at q<0.05, χ^2^. Thus, if accepting all 13 QTLs as true positives, it is simultaneously assumed that on average 5% of these would be false positives.

According to the QTLs, neither trait is controlled by a single gene or locus, but significantly affected by at least two to four regions of the genome. No overlap between QTLs for the separate traits was identified which suggests that the separate traits measure different aspects of host resistance. The percentages of phenotypic variation explained (PVE) levels detected for individual QTLs (4.6–10.1%), or combined for each trait (12–25.7%), suggest that the measured traits are complex in nature and probably controlled by a large number of loci. This is expected as defence against a necrotroph such as *H. parviporum* is not based on gene-for-gene interactions, but rather on the employment of a large battery of genes involved in systemic resistance. Similar PVE levels were found for the respective loci associated with bud set and height growth in a 283 individual population of *P. mariana*; 4–11.7% and 6.5–12.3% [61]. In a composite *P. glauca* map, two populations of 500 respective 200 individuals were used to detect QTLs for bud flush, bud set and height growth, explaining at the most 16.4, 22.2 and 10.5% of the respective variation of these traits [62], As for other conifers, the PVE of height growth, wood density and fibre length QTLs in *Pseudotsuga menziesii* peaked at 17.7, 14.9 and 15.7% [63] in a population of 320 trees (40 from each of 8 full-sib families), and resistance to *Dothistroma* needle blight in *Pinus radiata,* using 202 individuals from 6 full-sib families,was only explained to 4.8% by the strongest loci [12].

In every QTL mapping experiment, there is a danger of overestimating the importance of identified QTLs due to the so called Beavis effect [64]. According to Beavis, undetected QTLs with small effects inflate the estimated impact of closely located, detected QTLs. This bias has been reported to increase with LOD significance threshold and decrease with population size and map saturation [65]. Typically, large PVE-values in low density maps and small mapping populations run a greater risk of being affected by the Beavis effect. Indeed, Pelgas and coworkers [62] compared results from two mapping populations of 260 and 500 progenies respectively. They found that only 24% of the QTLs found in the larger population were also identified in the smaller, and that a randomized subset of 250 progenies out of the 500 only identified 29% of the QTLs. Furthermore, the PVE values of QTLs in the smaller population were twice as large as those obtained with the larger. This suggests that our population of 247 individuals was not large enough to disregard the Beavis effect and high PVE values should be regarded with caution as they might be the result of overestimation. However, this effect is a lesser factor for QTLs of lower PVE values. Jermstad et al [66]–[67] found that expanding a population from 98 to about 400 only changed the PVE interval for identified QTLs from 1.2–11.5% to 0.7–9.5%. Since all QTLs identified in this study fall within a similar range (PVE 4.6–10.1%), it is conceivable that the Beavis effect has played a minor role in determining their PVE.

When screening the infected trees for *H. parviporum* conidiophores, Arnerup *et al*. [24] concluded that 27% of the ramets contained no fungi. The reasons for this could be either technical, for example poorly infested wood blocks, or biological, i.e. some inherent trait that allows the host to prevent the establishment of the fungus in the wound. By mapping the proportion of immune ramets of each progeny as a trait, we discovered that 18.4% of this variation was explained by three QTLs on LG 1, 2 and 11 (Table 1). This result suggests that infection prevention at a very early stage is a heritable trait. As such a trait could potentially confer complete resistance to infection, rather than just limit the fungal spread within the tree, these QTLs might be of special interest for future *P. abies* breeding programmes.

The size of the *P. abies* genome, the low density of coding regions per MB and the difficulties with assembling genomes rich in transposable elements will complicate the investigation of the whole sequence corresponding to each QTL. In fact, as each assembled scaffold contains only one gene or less on average, we can at present only identify candidate genes from the QTLs if the map carries a SNP marker from them. The present linkage map contains 686 gene-based markers, a mere 2.4%, of the 28,354 genes of the *P. abies* genome [11]. However, some of the SNPs within the QTLs do originate from genes with known functions in plant response to wounding or microbial infection and have previously been shown to be regulated during *H. parviporum* infection or belong to the same biosynthethic pathways. This warrants discussing them, despite the low proportion of represented genes.

Lignin plays an important part in both chemical and mechanical resistance against pathogens on *P. abies* [68]. Several of the SNPs found within the QTL confidence intervals are known intermediates in the lignification process. The QTL on LG 11 contained, at 114.3 cM, a SNP originating from the transcription factor R2R3-MYB11. The R2R3-MYB transcription factor family plays a role in lignin metabolism [42], and transcripts of the *MYB11* gene were shown to increase slightly but significantly in the lignin enriched compressed wood of *P. glauca* compared to opposite wood [69]. One of the other QTLs for infection prevention, at 158.4 cM on LG 1, contained a SNP from a gene for hydroxycinnamoyl CoA shikimate/quinate hydroxycinnamoyltransferase (HCT). HCT has been shown to be involved in lignin biosynthesis in several plants, including *Pinus radiata* [40] and *Populus euramericana* [70]. Furthermore, a SNP from a gene for 4-coumarate CoA-ligase (4CL) was located at 76.0 cM on LG 3, in a QTL for the fungal exclusion trait. This enzyme is involved in the conjugation of hydroxycinnamates with CoA, and thus also related to the biosynthesis of phenyl propanoid [41].

Another interesting observation was made in the QTL for fungal growth within sapwood on LG 6. A SNP from a gene for leucoanthocyanidin reductase (accession number BT10950 in Table 1, *LAR1* in [23]) was mapped to 182.6 cM. *LAR1* is an important intermediate in the flavonoid pathway, converting leucoanthocyanidins to catechins, which subsequently are converted into the defence-related phenolics flavonoid-derived proanthocyanidins [71]. Both catechin and *LAR1* mRNA-levels, as well as other intermediates in the flavonoid pathway, have previously been shown to significantly increase in *P. abies* clones in response to *Heterobasidion*. The increase was greater in less susceptible clones than in more susceptible [24], [72]. Still, this pathway is not unique for reactions against *Heterobasidion* attack but it is rather involved in general host response to many kinds of stress. For example, the very same *LAR* gene identified in this study is also induced in *P. abies* in response to *Ceratocystis polonica* inoculation and wounding (*LAR3* in [71]). Taken together, this strengthens the notion that the BT10950 locus is important for *P. abies* resistance to *H. parviporum* infections.

In this study, we used 247 full-sib progenies and 686 SNP markers derived from transcribed genes to construct one of the most saturated, and the most gene-enriched, *P. abies* maps to date. It constitutes a powerful tool for future mapping of important traits and understanding of recombination events in conifers. We used the map to identify QTL regions for four distinct resistance traits involved in host response to *H. parviporum* infection, explaining in total 12–25.7% of the variation for each trait. This showed that all traits are complex and controlled by several key genes on different chromosomes, as would be expected when measuring systemic resistance. When analysing the SNP markers within the QTLs, we identified four genes previously reported to play a part in plant defence against fungal attack, of which one has previously been shown to be regulated during infection and three play a role in the phenyl propanoid pathway. Albeit the significant SNPs likely represent only a few of the genes present in the sequence covered by the QTLs, they can be used as valuable starting points for further investigations.

## Supporting Information

Table S1 **SNP information.** Short name, full name, accession information for GenBank and TAIR databases and from the *Picea glauca* genome, and full nucleotide sequence, for all 874 SNPs polymorphic between the parental trees.
(XLSX)

Table S2 **JoinMap files.** Input files used to compute the linkage map using JoinMap 3.0.
(TXT)

Table S3 **MapQTL files.** Input files used to compute the QTL mapping using MapQTL 4.0.
(TXT)
